# Sensory functions and their relation to balance metrics: a secondary analysis of the LIMBIC-CENC multicenter cohort

**DOI:** 10.3389/fneur.2023.1241545

**Published:** 2023-09-14

**Authors:** Susanne M. van der Veen, Robert Perera, Peter C. Fino, Laura Manning Franke, Amma A. Agyemang, Karen Skop, Elisabeth A. Wilde, Scot R. Sponheim, Alexander Stamenkovic, James S. Thomas, William C. Walker

**Affiliations:** ^1^Department of Physical Therapy, Virginia Commonwealth University, Richmond, VA, United States; ^2^Department of Physical Medicine and Rehabilitation, Virginia Commonwealth University, Richmond, VA, United States; ^3^Department of Biostatistics, Virginia Commonwealth University, Richmond, VA, United States; ^4^Department of Health and Kinesiology, University of Utah, Salt Lake City, UT, United States; ^5^Department of Physical Medicine and Rehabilitation Services, James A. Haley Veterans’ Hospital, Tampa, FL, United States; ^6^Department of Physical Medicine and Rehabilitation, Michael E. DeBakey VA Medical Center, Houston, TX, United States; ^7^Baylor College of Medicine, Houston, TX, United States; ^8^Department of Neurology, University of Utah, Salt Lake City, UT, United States; ^9^Minneapolis VA Health Care System, Minneapolis, MN, United States; ^10^Department of Psychiatry and Behavioral Sciences, University of Minnesota, Minneapolis, MN, United States; ^11^Hunter Holmes McGuire Veterans Affairs Medical Center, Richmond, VA, United States

**Keywords:** TBI, balance, sensory functions, auditory, vision

## Abstract

**Introduction:**

Among patients with traumatic brain injury (TBI), balance problems often persist alongside hearing and vision impairments that lead to poorer outcomes of functional independence. As such, the ability to regain premorbid independent gait may be dictated by the level of sensory acuity or processing decrements that are shown following TBI assessment. This study explores the relationships between standardized sensory acuity and processing outcomes to postural balance and gait speed.

**Methods:**

Secondary analysis was performed on the Long-Term Impact of Military- Relevant Brain Injury Consortium Chronic Effects of Neurotrauma Consortium LIMBIC (CENC) data set. Separate regression analyses were carried out for each of the balance assessments (via Computerized Dynamic Posturography, CDP) and walking speed.

**Discussion:**

TBI frequency was significantly related to the majority of single CDP outcomes (i.e., Conditions 2–6), while various sensory processing outcomes had task-specific influences. Hearing impairments and auditory processing decrements presented with lower CDP scores (CDP Conditions 3,5,6, and 1–3 respectively), whereas greater visual processing scores were associated with better CDP scores for Conditions 2,5, and 6. In sum, patients with TBI had similar scores on static balance tests compared to non-TBI, but when the balance task got more difficult patients with TBI scored worse on the balance tests. Additionally, stronger associations with sensory processing than sensory acuity measures may indicate that patients with TBI have increased fall risk.

## 1. Introduction

Patients with traumatic brain injury (TBI) often have chronically persisting symptoms of dizziness, nausea, and postural instability. This includes patients with mild TBI, in whom balance and gait problems can persist for more than 3 months (1–8), especially when caused by blast exposure (e.g., military injury, industrial accidents) (9). Besides balance impairments, blast-related TBI has been associated with a loss of hearing (19%), vision (34%) or both (32%) in a TBI population admitted to a Veterans Affairs (VA) Polytrauma Rehabilitation Center (PRC). When both hearing and vision are impaired, poorer functional independence at discharge has been reported (10). Critically, this is independent of TBI severity. The ability to regain premorbid balance and independent gait may be dictated by the ability to process, interpret, and combine, sensory information. Specifically, gait speed and the ability to maintain balance may be dictated by the perceived sensory information and subsequent sensorimotor integration and motor transformation necessary for successful task execution (11).

Postural control (whether for balance or gait purposes) depends on the integration of information from visual, vestibular, and somatosensory systems (12). In healthy individuals, the weighting of each sensory input adjusts to a decrease or loss in quality from any one input to preserve balance and maintain postural stability (13), and optimize movement efficiency (12). For example, while vision is an important sensory system used to maintain optimal postural stability (14), when visual information is occluded (c.f., closing your eyes) the CNS can adapt the weighting of the visual system, and upregulate the sensitivity of the vestibular and somatosensory inputs to maintain balance (13). However, during more complex (and dynamic) tasks, integration informed by all sensory inputs may be more critical to task success. When walking in cluttered terrain, where multiple obstacles complicate foot-placement (15), visual information can be leveraged in a feed-forward manner to register (1) where the foot needs to be placed safely and (2) ongoing visual monitoring of the foot to safely place the foot.

While few studies have investigated how impaired sensory systems affect the mobility of patients post-TBI, we can gather insights from the known influences to balance [including increased fall risk (16)] and deterioration in gait speed and performance that occur with sensory decline as a function of aging (17, 18). It is well known though that eyesight, hearing, vestibular function (17), and proprioception (18) all decline with age. While evidence indicates that the decline in sensory systems may play a role in the increase of fall risk (16) and deterioration of gait speed, these relationships have not been extensively studied. In the general population, the elderly rely more on their visual system to maintain postural stability, and gait is slower and more variable when the visual system is perturbed (19). Further, visual acuity, contrast sensitivity, and stereo acuity were also associated with greater risk of walking limitations during a 5-year follow-up (20). Finally, impaired hearing is reported to be related to a slower maximal gait speed, self-reported walking difficulties (19), and postural stability (21, 22). This relationship between hearing and gait speed and balance may be explained by the information hearing provides of our surroundings and/or because the vestibular organs share structure and function: they are anatomically closely localized, share fluid-filled bony compartments and blood circulation, are both served by the eighth cranial nerve, and have similar mechanosensory receptor hair cells, which detect sound, head movements, and orientation in space. However, all these findings are in the aging population in general, and it is largely unknown how sensory decline and balance, and mobility impairments are related to central nervous system deficits due to TBI.

Therefore, the aim of this study was to determine relationships amongst balance, gait, and sensory measures in a large cohort study including patients with one or more mild TBIs. It is hypothesized that the quality of gait and balance decline as the number and severity of sensory impairments increase.

## 2. Methods

### 2.1. Design

The study utilized an observational design with cross-sectional analyses using hierarchical regression to examine the predictive value of sensory measures of hearing and vision including auditory and visual processing measures on gait and balance.

Methods are described in more detail in van der Veen et al. (23).

### 2.2. Outcome measures

#### 2.2.1. Sensory-specific balance assessment (via CDP scores)

**Figure 1:**
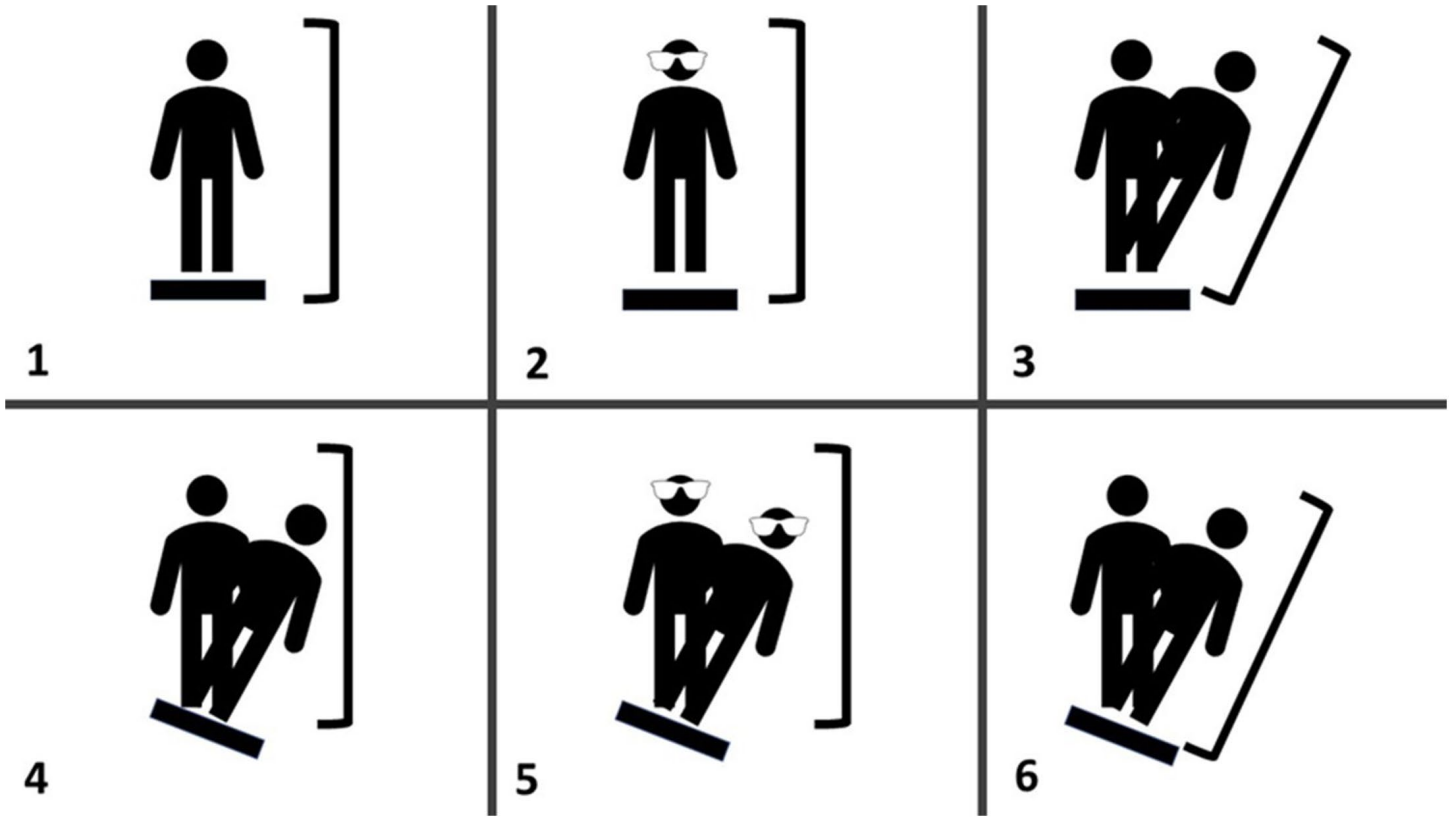
Schematic representation of the CDP the various panels represent the different balance assessments; (1) eyes open with fixed surface and surroundings, (2) eyes closed with fixed surface, (3) eyes open with fixed surface and sway-referenced visual surround, (4) eyes open with sway-referenced surface and fixed visual surround, (5) eyes closed with a sway-referenced surface, and (6) eyes open with sway-referenced surface and visual surround.

The computerized dynamic posturography (CDP) protocol on the NeuroCom Smart Balance Master (previously Natus, Inc) was used to assess postural balance. An embedded dual-plate force platform was used to generate equilibrium scores; ranging from 0 (touching a support surface, shifting feet, or falling) to 100 (little or no sway) for six sensory conditions: (1) all sensory inputs available; (2) no visual feedback; (3) distorted visual feedback because visual surround is “center of pressure referenced” (movements are proportional to the anterior–posterior displacement of the COP); (4) distorted somatosensory feedback because supporting platform is “center of pressure referenced”; (5) same as condition 4, but now with eyes closed; and (6) distorted visual and somatosensory feedback because both visual surround and supporting platform are “center of pressure referenced” (Figure 1). Each subject performed three trials for each condition, with an overall Composite CDP score calculated as a weighted average of the 6 scores (i.e., conditions 1 and 2 are weighted 1/3 as much as conditions 3 through 6).

#### 2.2.2. Walking speed

Gait was measured as part of the NIH Toolbox by the 4-meter walk score representing gait speed (24). This test is adapted from the 4-meter walk test in the short physical performance battery, an assessment tool for evaluating lower extremity functioning in older persons. Participants were asked to walk 4 meters at their usual pace twice, both attempts were timed in seconds, with the better trial used for scoring (calculation to walking speed in m/s).

### 2.3. Sensory tests

#### 2.3.1. Corrected visual acuity

Visual acuity is a measure determining clarity of vision with the subject standing 20 feet from the Snellen Eye Chart and the distance at which the participant can read the line of letters (25). If the participant normally wears glasses or contact lenses, the test was performed while wearing glasses or contacts. A left and right visual acuity score was measured and a threshold score for the right eye was met with a visual acuity score of 20/40.

#### 2.3.2. Visual spatial memory

The brief visuospatial memory test-revised (BVMT-R) is a measure of immediate and delayed visual memory (26). It requires the participant to reproduce line figures from memory. The BVMT-R provides twelve scores; three recall performance scores, one for each trial; a delayed recall score; three memory summary scores; three summary learning scores; hits (number of correct ‘yes’ responses) during the delayed recognition tasks; and a false alarm score (number of incorrect ‘yes’ responses) during the delayed recognition task.

#### 2.3.3. Auditory processing

The Scan-3 test is comprised of a screening battery of tests to detect auditory processing disorders in adolescents and adults (27). The test evaluates temporal processing with three subtests: gap detection; auditory figure ground; and competing words.

#### 2.3.4. Hearing handicap

The hearing handicap inventory for adults (HHIA) is a well-studied and widely used self-report measure of the respondent’s perceived hearing difficulty (28). The 11-item screening version used in this study is composed of two subscales (emotional and situational).

### 2.4. Data analysis

**Figure 2:**
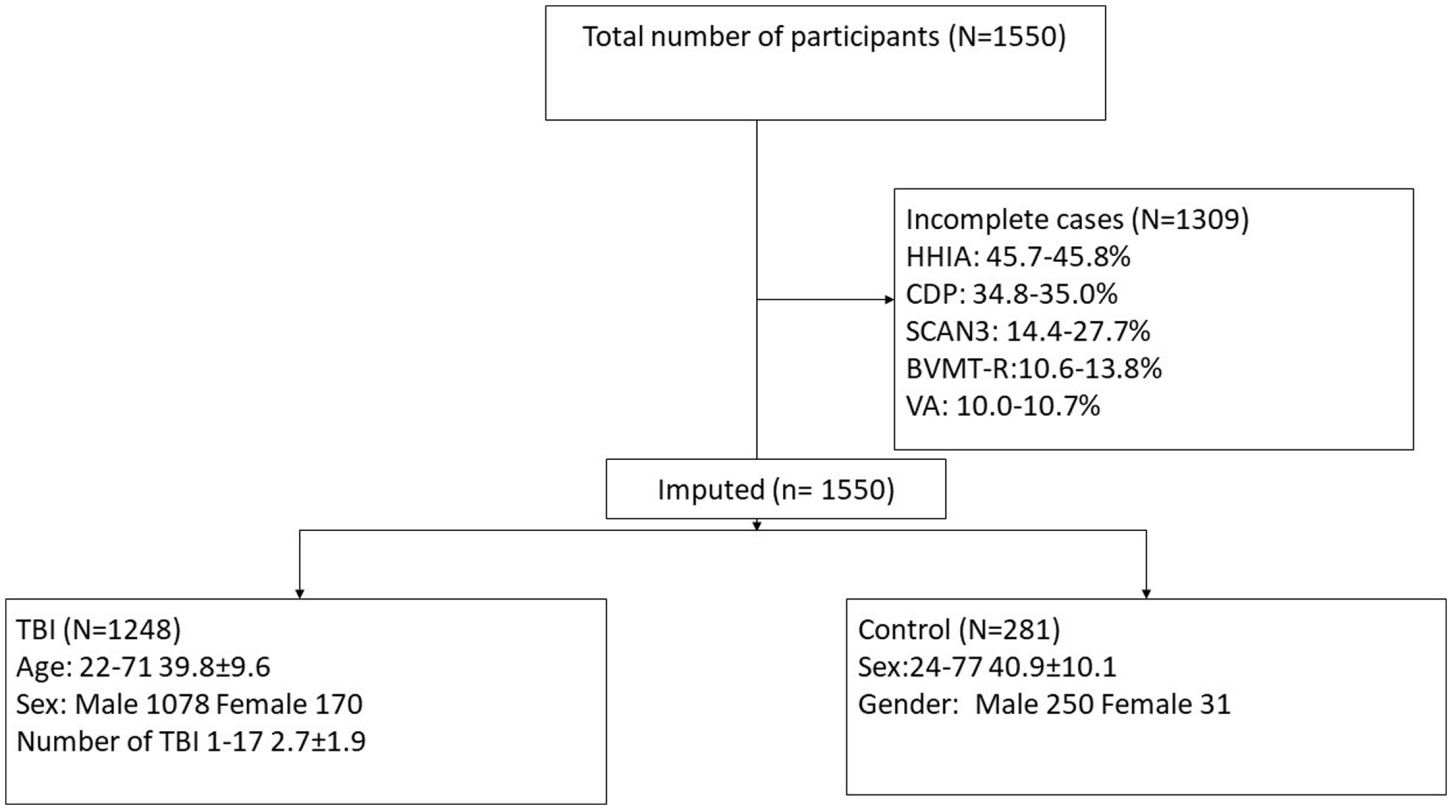
Consort diagram demonstrating participant selection for the current study. HHIA, Hearing Handicap Inventory for Adults; CDP, Computerized Dynamic Posturography; SCAN-3, Tests for Auditory Processing Disorders; BVMT-R, Brief Visuospatial Memory Test-Revised; VA, Visual acuity; TBI, traumatic brain injury.

Participant characteristics were summarized using means and standard deviations or frequencies (see Table 1). Missing data was accounted for using multiple imputation using SPSS (IBM Corp. Released 2020. IBM SPSS Statistics for Windows, Version 27.0. Armonk, NY: IBM Corp), see percentages in Figure 2. Five imputed datasets were created using a fully condition specification. The estimates were then combined, and standard errors were adjusted to account for the uncertainty due to missingness. Hierarchical regressions were performed using SPSS (IBM Corp. Released 2020. IBM SPSS Statistics for Windows, Version 27.0. Armonk, NY: IBM Corp) with TBI classification and covariates of interest grouped in the following 5 steps: (1) the number of TBIs suffered, age, and sex, (2) the separate HHIA items, (3) separate BVMT items, (4) visual acuity, and (5) items of the SCAN3 (see Table 2 for a complete overview of the items entered in the regression). Separate hierarchical regression analyses were carried out for each of the balance assessment outcome measures (i.e., best 4 m walk score, CDP composite, CDP condition 1–6). Sensory measures were removed from the regression equations when collinearity was found (VIF > 10). Statistical significance was determined using a Benjamini-Hochberg correction, where the critical *p* values were based on the 27 tests per regression and a fall discovery rate of 20%.

**Table 1:** Participant demographics, mean ± standard deviation, except for sex male, feale.

	TBI		Control		Total	
	Mean	Std	Mean	Std	Mean	Std
Age (mean/std)	39.93	9.57	40.01	10.08	39.95	9.66
Sex (male/female)	1126	140	221	63	1347	
TBI	2.7	1.93	0	0	2.2	2.03
walking speed (m/s)	1.24	0.37	1.2	0.22	1.24	0.35
CDP composite	72.63	13.78	74.7	8.92	73.01	13.09
CDP1	92.43	5.05	92.76	4.06	92.5	7.00
CDP2	88.19	7.44	89.87	3.9	88.5	9.34
CDP3	86.7	9.99	88.88	4.63	87.1	17.12
CDP4	59.14	18.01	76.09	11.7	73.76	19.22
CDP5	58.67	20.03	61.6	14.57	59.59	21.91
CDP6	72.63	22.61	60.57	18.13	59.02	19.09
Visual acuity right	1.00	0.34	1.00	0.34	1.00	0.34
Visual acuity left	1.03	0.33	1.05	0.35	1.03	0.34
Scan-3	0.47	0.50	0.49	0.50	0.47	0.50
HHIA	16.45	8.53	15.21	7.11	16.26	8.33
BVMT-R mean recall	42.52	12.32	42.68	12.10	42.55	12.28
BVMT-R delayed recall	44.18	13.02	45.95	12.73	44.51	12.98
BVMT-R mean learning	51.66	11.74	53.55	10.74	52.01	11.20

**Table 2:** Results from the hierarchical regression for best walk score.

WALK score (m/s)	Model 1				Model 2				Model 3				Model 4				Model 5			
	*B*	Std. error	*t*	*p*	*B*	Std. error	*t*	*p*	*B*	Std. error	*t*	*p*	*B*	Std. error	*t*	*p*	*B*	Std. error	*t*	*p*
*R* ^2^	0.021				0.052				0.074				0.130				0.157			
Constant	1.176	0.050	23.325	0.000	1.176	0.069	17.029	0.000	1.161	0.123	9.441	0.000	0.791	0.142	5.573	0.000	0.757	0.148	5.099	0.000
DEMOGAGEYEARS	0.002	0.001	1.661	0.097	0.002	0.001	1.964	0.050	0.003	0.001	2.655	0.008	0.004	0.001	3.372	0.002	0.003	0.001	2.695	0.010
GENDERTYP	−0.008	0.027	−0.291	0.771	−0.005	0.027	−0.195	0.845	−0.010	0.028	−0.370	0.712	−0.006	0.026	−0.244	0.808	−0.024	0.026	−0.934	0.350
TOTAL_TBI	0.002	0.004	0.525	0.600	0.003	0.005	0.680	0.497	0.003	0.005	0.594	0.552	0.000	0.005	0.068	0.946	0.000	0.005	0.064	0.949
HHIASEMBARRASSEDNEWPEOPLE					−0.006	0.005	−1.318	0.191	−0.006	0.005	−1.307	0.194	−0.006	0.004	−1.285	0.201	−0.005	0.004	−1.231	0.221
HHIASFEELFRUSTRATED					0.004	0.011	0.354	0.727	0.004	0.011	0.346	0.734	0.004	0.011	0.341	0.737	0.004	0.011	0.374	0.714
HHIASDIFFICULTYUNDERSTANDING					−0.005	0.010	−0.502	0.617	−0.006	0.011	−0.533	0.597	−0.009	0.010	−0.879	0.384	−0.010	0.010	−1.040	0.303
HHIASFEELHANDICAPPED					−0.003	0.009	−0.373	0.709	−0.002	0.009	−0.179	0.858	0.001	0.009	0.065	0.948	0.000	0.009	−0.026	0.980
HHIASDIFFICULTYVISITING					0.001	0.011	0.080	0.937	0.001	0.011	0.103	0.919	−0.001	0.011	−0.102	0.920	−9.948E-05	0.011	−0.009	0.993
HHIASDIFFICULTYINMOVIES					−0.025	0.011	−2.227	0.039	−0.026	0.012	−2.259	0.039	−0.023	0.011	−2.031	0.061	−0.021	0.010	−2.138	0.043
HHIASARGUMENTSFAMILY					0.005	0.009	0.539	0.598	0.006	0.010	0.597	0.561	0.004	0.010	0.392	0.702	0.004	0.009	0.403	0.693
HHIASDIFFICULTYLISTENINGTV					−0.002	0.009	−0.258	0.798	−0.003	0.009	−0.326	0.746	−0.005	0.009	−0.567	0.573	−0.006	0.008	−0.714	0.477
HHIASHAMPERSPERSONALLIFE					0.006	0.010	0.567	0.575	0.006	0.011	0.560	0.581	0.007	0.011	0.633	0.536	0.006	0.010	0.621	0.541
HHIASDIFFICULTYRESTAURANT					0.003	0.009	0.292	0.771	0.002	0.009	0.192	0.848	0.002	0.008	0.204	0.839	0.003	0.008	0.415	0.678
HHIASPROBLEMWITHHEARING					0.007	0.021	0.356	0.723	0.009	0.021	0.449	0.654	0.004	0.021	0.174	0.862	−0.010	0.022	−0.469	0.641
BVMTRRECALLTSCORE									0.003	0.001	2.292	0.022	0.003	0.001	2.452	0.016	0.003	0.001	2.589	0.011
BVMTRLEARNINGTSCORE									−0.001	0.001	−0.759	0.448	1.079E-05	0.001	0.011	0.991	0.000	0.001	−0.351	0.726
BVMTRDELAYEDRECALLTSCORE									−0.004	0.001	−2.993	0.003	−0.004	0.001	−3.147	0.002	−0.003	0.001	−2.711	0.008
BVMTRHITRAWSCORE									0.008	0.038	0.200	0.842	0.000	0.036	0.006	0.995	−0.001	0.036	−0.025	0.980
BVMTRFALSEALARMRAWSCORE									−0.065	0.041	−1.583	0.114	−0.049	0.041	−1.178	0.239	−0.043	0.040	−1.068	0.286
BVMTRDISCRIMINTATIONRAWSCORE									0.005	0.033	0.156	0.876	0.007	0.032	0.223	0.823	0.011	0.031	0.347	0.729
VA_RT_score													0.056	0.068	0.829	0.430	0.044	0.066	0.669	0.522
VA_RT_inter													0.116	0.120	0.972	0.370	0.109	0.112	0.976	0.367
VA_LT_score													0.151	0.088	1.716	0.137	0.148	0.087	1.692	0.142
VA_LT_inter													0.014	0.194	0.071	0.946	0.004	0.180	0.020	0.985
SCAN3GAPDETECTGRADE																	0.062	0.037	1.665	0.102
SCAN3AUDITFIGURECOMBINEDSCORE																	0.012	0.002	6.340	0.000
SCAN3COMPETEWORDCOMBINEDSCORE																	−0.011	0.002	−5.550	0.000

## 3. Results

### 3.1. Participants

The study includes data from 1550 participants, but only 241 (15.55%) cases were complete (see Figure 2). All participants were included in analyses due to the use of multiple imputation. Of these 1550, 1248 suffered at least one TBI and 281 were participants with no history of TBI (non-TBI). For demographics, see Table 1.

### 3.2. Walking speed

Table 2 presents the complete hierarchical regression results for the 4 m walking speed. Step 2 revealed a negative association between the difficulty with the item “understanding movies” (*p* = 0.012), “problems with hearing” (*p* = 0.003), and walk score. Step 3 indicated an increase in valid items recalled after a delay was associated with slower walking speeds; age (*p* = 0.006) became related to faster walking speeds. Although Step 4 (visual acuity added) increased variance accounted for to 12.0%, none of the visual acuity measures were significant; visual spatial recall memory (BVMT-R delayed recall score, *p* = 0.013) remained positively related to 4 m walk time. Step 5 added audio processing and increased variance accounted for to 14.0%. The ability to distinguish audio target from noise showed a relation with faster walking speeds (*p* = 0.001), indicating the ability to distinguish words from noise was related to longer 4 m walk times. See Table 2 for the complete results.

### 3.3. CDP composite

Table 3 presents the complete hierarchical regression results for the CDP composite score. Step 1 accounted for 6.2% of the variance of the composite CDP score. All demographic measures were found to be related to balance measured with the CDP combined score. Age, sex, and number of TBI are negatively correlated with the CDP composite score, indicating older people (*p* = 0.039), females (*p* = 0.045), and people with more TBIs suffered (*p* < 0.001) have more balance difficulties. Step 2, revealed an association between self-reported absence of difficulty with hearing (*p* = 0.001) and a better CDP composite score. Step 3 showed visual spatial recall memory (BVMT-R delayed recall score, *p* = 0.014) was positively related to the CDP composite score. A positive relationship was shown between auditory processing [the ability to distinguish audio target from noise (*p* = 0.034) and the ability to repeat both words (*p* = 0.020)] and CDP composite score in step 5. See Table 3 for the individual measures.

**Table 3:** Results from the hierarchical regression for CDP composite.

CDP composite	Model 1				Model 2				Model 3				Model 4				Model 5			
	*B*	Std. error	*t*	*p*	*B*	Std. error	*t*	*p*	*B*	Std. error	*t*	*p*	*B*	Std. error	*t*	*p*	*B*	Std. error	*t*	*p*
*R* ^2^	0.062				0.094				0.118				0.124				0.138			
Constant	79.471	1.925	41.286	0.000	76.658	2.638	29.061	0.000	66.965	3.970	16.869	0.000	62.879	4.383	14.347	0.000	53.958	4.876	11.066	0.000
DEMOGAGEYEARS	−0.074	0.035	−2.123	0.039	−0.065	0.036	−1.796	0.081	−0.040	0.037	−1.086	0.286	−0.030	0.040	−0.737	0.469	−0.001	0.040	−0.013	0.990
GENDERTYP	−1.811	0.901	−2.010	0.045	−2.262	0.870	−2.599	0.009	−2.411	0.863	−2.795	0.005	−2.402	0.858	−2.800	0.005	−2.386	0.854	−2.796	0.005
TOTAL_TBI	−0.654	0.154	−4.243	0.000	−0.488	0.166	−2.933	0.005	−0.525	0.166	−3.160	0.003	−0.545	0.165	−3.297	0.002	−0.558	0.162	−3.444	0.001
HHIASEMBARRASSEDNEWPEOPLE					−0.103	0.201	−0.512	0.618	−0.091	0.203	−0.445	0.665	−0.085	0.200	−0.427	0.678	−0.056	0.204	−0.274	0.789
HHIASFEELFRUSTRATED					0.075	0.356	0.212	0.835	0.104	0.344	0.303	0.765	0.097	0.340	0.285	0.779	0.060	0.332	0.181	0.858
HHIASDIFFICULTYUNDERSTANDING					0.035	0.387	0.091	0.929	0.053	0.373	0.141	0.889	0.004	0.378	0.012	0.991	0.069	0.363	0.189	0.852
HHIASFEELHANDICAPPED					−0.235	0.325	−0.723	0.473	−0.128	0.311	−0.411	0.682	−0.095	0.309	−0.306	0.760	−0.059	0.308	−0.190	0.850
HHIASDIFFICULTYVISITING					−0.090	0.452	−0.198	0.847	−0.122	0.460	−0.265	0.797	−0.152	0.464	−0.327	0.750	−0.168	0.473	−0.356	0.730
HHIASDIFFICULTYINMOVIES					0.094	0.323	0.292	0.772	0.110	0.325	0.337	0.739	0.138	0.319	0.433	0.669	0.153	0.311	0.493	0.625
HHIASARGUMENTSFAMILY					−0.255	0.284	−0.899	0.380	−0.246	0.260	−0.948	0.351	−0.264	0.261	−1.010	0.322	−0.201	0.265	−0.761	0.454
HHIASDIFFICULTYLISTENINGTV					−0.089	0.414	−0.214	0.835	−0.120	0.408	−0.295	0.774	−0.135	0.403	−0.336	0.744	−0.063	0.379	−0.165	0.872
HHIASHAMPERSPERSONALLIFE					−0.381	0.370	−1.030	0.319	−0.281	0.355	−0.792	0.439	−0.265	0.357	−0.744	0.467	−0.233	0.348	−0.671	0.510
HHIASDIFFICULTYRESTAURANT					−0.106	0.318	−0.333	0.741	−0.184	0.333	−0.552	0.586	−0.182	0.351	−0.519	0.610	−0.160	0.346	−0.464	0.648
HHIASPROBLEMWITHHEARING					2.721	0.750	3.627	0.001	2.624	0.755	3.475	0.002	2.517	0.762	3.303	0.003	1.974	0.730	2.703	0.010
BVMTRRECALLTSCORE									0.096	0.039	2.459	0.014	0.098	0.040	2.480	0.014	0.084	0.040	2.115	0.036
BVMTRLEARNINGTSCORE									−0.042	0.028	−1.512	0.131	−0.035	0.028	−1.262	0.207	−0.040	0.028	−1.427	0.153
BVMTRDELAYEDRECALLTSCORE									0.008	0.037	0.224	0.823	0.004	0.037	0.106	0.916	0.004	0.037	0.104	0.917
BVMTRHITRAWSCORE									1.372	1.175	1.167	0.243	1.271	1.192	1.066	0.287	1.186	1.187	0.999	0.318
BVMTRFALSEALARMRAWSCORE									−1.123	1.285	−0.874	0.382	−1.039	1.284	−0.809	0.418	−0.857	1.284	−0.667	0.505
BVMTRDISCRIMINTATIONRAWSCORE									−0.190	1.060	−0.180	0.857	−0.159	1.066	−0.149	0.881	−0.101	1.072	−0.094	0.925
VA_RT_score													0.381	1.377	0.277	0.783	0.453	1.381	0.328	0.744
VA_RT_inter													3.145	2.878	1.093	0.306	2.834	2.852	0.994	0.349
VA_LT_score													0.928	1.572	0.590	0.561	0.656	1.580	0.415	0.682
VA_LT_inter													−0.270	3.366	−0.080	0.938	0.010	3.307	0.003	0.998
SCAN3GAPDETECTGRADE																	−0.371	1.158	−0.321	0.749
SCAN3AUDITFIGURECOMBINEDSCORE																	0.157	0.071	2.205	0.034
SCAN3COMPETEWORDCOMBINEDSCORE																	0.195	0.079	2.471	0.020

### 3.4. CDP condition 1 eyes open with fixed surface and visual surround

Table 4 represents the complete hierarchical regression results for the CDP condition 1 score. Step 1 accounted for 2.1% of the variance of the CDP condition 1 score. In step 2, an association between increased difficulty understanding new people (*p* = 0.025) and worse CDP condition 1 score was found. In step 5 a relationship was shown between auditory processing (the ability to repeat both words, *p* = 0.001) and CDP condition 1. See Table 4 for the individual measures.

**Table 4:** Results from the hierarchical regression for CDP condition 1, standing balance.

CDP1 standing balance	Model 1				Model 2				Model 3				Model 4				Model 5			
	*B*	Std. error	*t*	*p*	*B*	Std. error	*t*	*p*	*B*	Std. error	*t*	*p*	*B*	Std. error	*t*	*p*	*B*	Std. error	*t*	*p*
*R* ^2^	0.021				0.053				0.083				0.087				0.109			
Constant	93.490	0.782	119.553	0.000	93.469	1.253	74.590	0.000	89.763	1.822	49.272	0.000	92.089	2.282	40.347	0.000	90.893	3.011	30.188	0.000
DEMOGAGEYEARS	−0.015	0.015	−0.987	0.328	−0.017	0.015	−1.096	0.278	−0.009	0.016	−0.561	0.577	−0.006	0.015	−0.404	0.687	0.007	0.015	0.481	0.631
GENDERTYP	−0.210	0.402	−0.522	0.602	−0.247	0.400	−0.616	0.538	−0.291	0.397	−0.732	0.465	−0.318	0.392	−0.813	0.417	−0.150	0.400	−0.374	0.709
TOTAL_TBI	−0.076	0.063	−1.213	0.225	−0.054	0.064	−0.835	0.404	−0.056	0.064	−0.888	0.375	−0.048	0.064	−0.739	0.460	−0.052	0.064	−0.804	0.422
HHIASEMBARRASSEDNEWPEOPLE					0.026	0.071	0.372	0.713	0.034	0.070	0.488	0.629	0.038	0.068	0.558	0.580	0.040	0.071	0.555	0.584
HHIASFEELFRUSTRATED					0.057	0.148	0.385	0.704	0.061	0.146	0.417	0.681	0.052	0.144	0.360	0.723	0.044	0.147	0.302	0.766
HHIASDIFFICULTYUNDERSTANDING					−0.303	0.133	−2.281	0.025	−0.297	0.128	−2.319	0.021	−0.278	0.130	−2.146	0.033	−0.258	0.127	−2.037	0.043
HHIASFEELHANDICAPPED					0.013	0.143	0.094	0.925	0.046	0.141	0.323	0.748	0.057	0.139	0.407	0.685	0.071	0.139	0.509	0.613
HHIASDIFFICULTYVISITING					−0.221	0.166	−1.330	0.199	−0.225	0.169	−1.330	0.201	−0.211	0.168	−1.250	0.228	−0.218	0.169	−1.288	0.216
HHIASDIFFICULTYINMOVIES					0.063	0.153	0.414	0.684	0.062	0.153	0.408	0.688	0.058	0.149	0.386	0.704	0.050	0.160	0.309	0.761
HHIASARGUMENTSFAMILY					0.002	0.108	0.023	0.982	0.013	0.105	0.126	0.900	0.017	0.105	0.160	0.874	0.030	0.107	0.283	0.778
HHIASDIFFICULTYLISTENINGTV					0.078	0.133	0.589	0.560	0.071	0.134	0.532	0.599	0.073	0.133	0.550	0.587	0.099	0.136	0.726	0.475
HHIASHAMPERSPERSONALLIFE					0.053	0.167	0.319	0.755	0.084	0.171	0.493	0.631	0.074	0.168	0.440	0.667	0.088	0.170	0.515	0.616
HHIASDIFFICULTYRESTAURANT					0.066	0.131	0.507	0.614	0.053	0.138	0.384	0.704	0.041	0.141	0.293	0.772	0.036	0.137	0.263	0.794
HHIASPROBLEMWITHHEARING					0.303	0.440	0.687	0.508	0.251	0.443	0.566	0.584	0.281	0.445	0.631	0.543	0.243	0.410	0.592	0.565
BVMTRRECALLTSCORE									0.008	0.019	0.404	0.688	0.009	0.019	0.488	0.627	0.004	0.019	0.231	0.818
BVMTRLEARNINGTSCORE									−0.024	0.014	−1.714	0.091	−0.025	0.014	−1.776	0.081	−0.024	0.015	−1.667	0.103
BVMTRDELAYEDRECALLTSCORE									0.013	0.017	0.722	0.472	0.012	0.018	0.695	0.489	0.008	0.018	0.426	0.671
BVMTRHITRAWSCORE									1.032	1.205	0.857	0.425	1.030	1.209	0.852	0.428	1.076	1.261	0.853	0.429
BVMTRFALSEALARMRAWSCORE									−0.930	1.013	−0.918	0.385	−0.976	1.039	−0.939	0.376	−0.988	1.077	−0.917	0.389
BVMTRDISCRIMINTATIONRAWSCORE									−0.364	1.091	−0.333	0.751	−0.370	1.101	−0.336	0.749	−0.422	1.145	−0.369	0.726
VA_RT_score													−0.131	0.608	−0.215	0.830	−0.059	0.632	−0.094	0.926
VA_RT_inter													−0.791	1.325	−0.597	0.569	−0.791	1.340	−0.590	0.573
VA_LT_score													0.611	0.581	1.052	0.295	0.617	0.571	1.079	0.282
VA_LT_inter													−2.230	2.001	−1.114	0.309	−2.080	1.894	−1.098	0.314
SCAN3GAPDETECTGRADE																	−1.104	0.853	−1.294	0.233
SCAN3AUDITFIGURECOMBINEDSCORE																	−0.048	0.048	−0.992	0.352
SCAN3COMPETEWORDCOMBINEDSCORE																	0.117	0.033	3.551	0.001

### 3.5. CDP condition 2 eyes closed with a fixed surface

Table 5 represents the complete hierarchical regression results for the CDP condition 2 score. Both Age (*p* = 0.010) and number of TBI (*p* = 0.001) were shown to be negatively related to CDP condition 2 score in step 1. Step 2, the absence of difficulty hearing (*p* = 0.013) was associated with a better CDP condition 2 score. Step 3 revealed a positive association was shown for the delayed recall score (*p* = 0.001). Step 4 showed visual learning score (*p* = 0.008) has a negative association with the CDP condition 2. Step 5 showed a positive relationship was shown between auditory processing [the ability to repeat both words (*p* < 0.001)] and CDP condition 2 scores. See Table 5 for the individual measures.

**Table 5:** Results from the hierarchical regression for CDP condition 2, occluded vision.

CDP2 no vision	Model 1				Model 2				Model 3				Model 4				Model 5			
	*B*	Std. error	*t*	*p*	*B*	Std. error	*t*	*p*	*B*	Std. error	*t*	*p*	*B*	Std. error	*t*	*p*	*B*	Std. error	*t*	*p*
*R* ^2^	0.068				0.096				0.122				0.125				0.144			
Constant	91.853	0.980	93.698	0.000	90.872	1.306	69.566	0.000	86.644	2.751	31.494	0.000	85.906	3.316	25.909	0.000	78.958	3.526	22.396	0.000
DEMOGAGEYEARS	−0.050	0.019	−2.611	0.010	−0.046	0.019	−2.383	0.018	−0.044	0.020	−2.156	0.034	−0.039	0.021	−1.901	0.061	−0.018	0.022	−0.799	0.430
GENDERTYP	−0.303	0.563	−0.538	0.592	−0.556	0.555	−1.001	0.318	−0.555	0.538	−1.032	0.303	−0.555	0.540	−1.029	0.304	−0.518	0.535	−0.968	0.334
TOTAL_TBI	−0.458	0.117	−3.911	0.001	−0.369	0.103	−3.589	0.001	−0.359	0.105	−3.423	0.002	−0.364	0.105	−3.465	0.001	−0.369	0.105	−3.521	0.001
HHIASEMBARRASSEDNEWPEOPLE					−0.003	0.098	−0.033	0.974	0.010	0.093	0.107	0.916	0.015	0.090	0.164	0.870	0.033	0.095	0.351	0.728
HHIASFEELFRUSTRATED					−0.064	0.259	−0.248	0.810	−0.061	0.243	−0.249	0.808	−0.066	0.244	−0.270	0.792	−0.101	0.250	−0.403	0.696
HHIASDIFFICULTYUNDERSTANDING					0.004	0.271	0.016	0.988	0.020	0.252	0.080	0.937	0.005	0.254	0.020	0.985	0.072	0.239	0.299	0.769
HHIASFEELHANDICAPPED					−0.111	0.252	−0.440	0.668	−0.084	0.244	−0.344	0.736	−0.073	0.241	−0.302	0.767	−0.048	0.235	−0.205	0.840
HHIASDIFFICULTYVISITING					−0.127	0.228	−0.557	0.584	−0.128	0.227	−0.564	0.580	−0.133	0.230	−0.577	0.571	−0.145	0.238	−0.608	0.552
HHIASDIFFICULTYINMOVIES					0.112	0.205	0.548	0.589	0.130	0.207	0.626	0.539	0.141	0.213	0.665	0.515	0.133	0.189	0.704	0.487
HHIASARGUMENTSFAMILY					−0.187	0.182	−1.024	0.324	−0.176	0.184	−0.957	0.356	−0.184	0.186	−0.989	0.341	−0.146	0.185	−0.788	0.446
HHIASDIFFICULTYLISTENINGTV					0.032	0.255	0.126	0.902	0.032	0.250	0.128	0.901	0.025	0.250	0.102	0.921	0.078	0.230	0.341	0.740
HHIASHAMPERSPERSONALLIFE					−0.063	0.258	−0.246	0.811	−0.020	0.257	−0.079	0.938	−0.016	0.258	−0.063	0.951	0.005	0.254	0.020	0.985
HHIASDIFFICULTYRESTAURANT					−0.224	0.225	−0.995	0.337	−0.228	0.228	−0.997	0.337	−0.226	0.232	−0.973	0.349	−0.223	0.226	−0.984	0.343
HHIASPROBLEMWITHHEARING					1.306	0.480	2.719	0.013	1.207	0.469	2.575	0.017	1.177	0.466	2.525	0.018	0.926	0.467	1.981	0.058
BVMTRRECALLTSCORE									−0.056	0.026	−2.198	0.031	−0.055	0.026	−2.121	0.038	−0.063	0.025	−2.588	0.011
BVMTRLEARNINGTSCORE									−0.049	0.018	−2.685	0.008	−0.047	0.019	−2.516	0.014	−0.048	0.019	−2.530	0.014
BVMTRDELAYEDRECALLTSCORE									0.080	0.024	3.277	0.001	0.078	0.025	3.115	0.003	0.077	0.025	3.099	0.003
BVMTRHITRAWSCORE									1.659	2.083	0.796	0.462	1.624	2.092	0.777	0.473	1.470	2.088	0.704	0.513
BVMTRFALSEALARMRAWSCORE									−1.330	1.666	−0.798	0.453	−1.290	1.672	−0.772	0.468	−1.182	1.691	−0.699	0.510
BVMTRDISCRIMINTATIONRAWSCORE									−0.701	1.931	−0.363	0.732	−0.693	1.925	−0.360	0.734	−0.610	1.932	−0.316	0.765
VA_RT_score													0.425	1.359	0.312	0.763	0.583	1.351	0.431	0.678
VA_RT_inter													0.193	2.052	0.094	0.928	−0.026	2.053	−0.013	0.990
VA_LT_score													0.380	1.202	0.316	0.758	0.142	1.181	0.121	0.906
VA_LT_inter													−0.302	1.527	−0.198	0.845	−0.033	1.458	−0.023	0.982
SCAN3GAPDETECTGRADE																	0.548	0.744	0.737	0.465
SCAN3AUDITFIGURECOMBINEDSCORE																	0.038	0.047	0.816	0.425
SCAN3COMPETEWORDCOMBINEDSCORE																	0.198	0.040	4.889	0.000

### 3.6. CDP condition 3 eyes open with fixed surface and sway-referenced visual surround

Table 6 represents the complete hierarchical regression results for the CDP condition 3 score. In step 1 number of TBI (*p* = 0.001) was shown to be negatively related to CDP condition 3 scores. Step 2 showed the absence of difficulty hearing (*p* = 0.004) was associated with a better CDP condition 3 score. A positive relationship was shown between auditory processing [the ability to repeat both words (*p* = 0.021) and CDP condition 3 score in step 5]. See Table 6 for the individual measures.

**Table 6:** Results from the hierarchical regression for CDP condition 3, sway referenced vision.

CDP3	Model 1				Model 2				Model 3				Model 4				Model 5			
	*B*	Std. error	*t*	*p*	*B*	Std. error	*t*	*p*	*B*	Std. error	*t*	*p*	*B*	Std. error	*t*	*p*	*B*	Std. error	*t*	*p*
*R* ^2^	0.078				0.109				0.184				0.128				0.138			
Constant	90.616	1.500	60.409	0.000	88.043	2.127	41.383	0.000	86.874	3.614	24.038	0.000	84.624	5.078	16.665	0.000	77.732	5.614	13.845	0.000
DEMOGAGEYEARS	−0.024	0.031	−0.789	0.439	−0.013	0.031	−0.427	0.674	−0.006	0.032	−0.192	0.850	−0.002	0.033	−0.058	0.955	0.016	0.035	0.456	0.655
GENDERTYP	−0.765	0.794	−0.963	0.341	−1.103	0.762	−1.448	0.152	−1.140	0.757	−1.506	0.137	−1.132	0.730	−1.551	0.124	−1.165	0.726	−1.604	0.111
TOTAL_TBI	−0.760	0.120	−6.362	0.000	−0.631	0.123	−5.123	0.000	−0.620	0.123	−5.018	0.000	−0.633	0.122	−5.175	0.000	−0.638	0.122	−5.243	0.000
HHIASEMBARRASSEDNEWPEOPLE					−0.002	0.162	−0.010	0.992	0.011	0.158	0.069	0.946	0.015	0.151	0.099	0.922	0.036	0.153	0.234	0.819
HHIASFEELFRUSTRATED					−0.028	0.226	−0.124	0.902	−0.027	0.220	−0.125	0.901	−0.034	0.222	−0.156	0.877	−0.067	0.228	−0.294	0.770
HHIASDIFFICULTYUNDERSTANDING					0.262	0.298	0.879	0.392	0.262	0.295	0.887	0.388	0.220	0.286	0.771	0.450	0.277	0.281	0.985	0.336
HHIASFEELHANDICAPPED					−0.292	0.243	−1.201	0.234	−0.265	0.248	−1.068	0.290	−0.270	0.244	−1.106	0.272	−0.255	0.242	−1.054	0.295
HHIASDIFFICULTYVISITING					−0.468	0.282	−1.659	0.110	−0.459	0.286	−1.604	0.123	−0.476	0.279	−1.705	0.101	−0.481	0.281	−1.713	0.100
HHIASDIFFICULTYINMOVIES					−0.148	0.261	−0.564	0.578	−0.145	0.261	−0.553	0.586	−0.123	0.270	−0.456	0.654	−0.122	0.264	−0.463	0.648
HHIASARGUMENTSFAMILY					−0.174	0.221	−0.790	0.440	−0.160	0.222	−0.720	0.481	−0.172	0.233	−0.739	0.472	−0.134	0.232	−0.580	0.571
HHIASDIFFICULTYLISTENINGTV					0.250	0.346	0.722	0.490	0.252	0.343	0.735	0.483	0.244	0.343	0.712	0.496	0.296	0.327	0.907	0.388
HHIASHAMPERSPERSONALLIFE					0.092	0.280	0.330	0.745	0.118	0.271	0.433	0.669	0.138	0.274	0.503	0.621	0.156	0.274	0.568	0.577
HHIASDIFFICULTYRESTAURANT					−0.322	0.272	−1.185	0.251	−0.320	0.278	−1.151	0.266	−0.296	0.305	−0.971	0.351	−0.285	0.299	−0.954	0.358
HHIASPROBLEMWITHHEARING					2.142	0.648	3.304	0.004	2.098	0.657	3.196	0.005	2.003	0.638	3.138	0.005	1.689	0.662	2.553	0.020
BVMTRRECALLTSCORE									−0.047	0.031	−1.503	0.134	−0.047	0.031	−1.538	0.125	−0.056	0.031	−1.771	0.078
BVMTRLEARNINGTSCORE									−0.057	0.025	−2.287	0.025	−0.055	0.026	−2.091	0.043	−0.057	0.026	−2.207	0.032
BVMTRDELAYEDRECALLTSCORE									0.038	0.031	1.237	0.218	0.038	0.031	1.231	0.220	0.039	0.031	1.239	0.218
BVMTRHITRAWSCORE									0.915	1.582	0.578	0.578	0.887	1.588	0.559	0.591	0.742	1.608	0.461	0.657
BVMTRFALSEALARMRAWSCORE									−1.895	1.431	−1.324	0.206	−1.809	1.454	−1.244	0.234	−1.671	1.461	−1.144	0.273
BVMTRDISCRIMINTATIONRAWSCORE									−0.177	1.460	−0.121	0.906	−0.188	1.447	−0.130	0.900	−0.096	1.460	−0.066	0.949
VA_RT_score													2.252	1.303	1.729	0.100	2.347	1.343	1.747	0.100
VA_RT_inter													−0.269	3.202	−0.084	0.936	−0.517	3.215	−0.161	0.878
VA_LT_score													−1.114	1.349	−0.826	0.421	−1.355	1.325	−1.022	0.322
VA_LT_inter													1.665	3.324	0.501	0.634	1.896	3.277	0.579	0.583
SCAN3GAPDETECTGRADE																	0.659	0.910	0.725	0.470
SCAN3AUDITFIGURECOMBINEDSCORE																	0.091	0.059	1.552	0.134
SCAN3COMPETEWORDCOMBINEDSCORE																	0.147	0.061	2.435	0.021

### 3.7. CDP condition 4 eyes open with sway-referenced surface and fixed visual surround

Table 7 represents the complete hierarchical regression results for the CDP condition 4 score. In step number of TBI (*p* = 0.006) showed a negative relation to CDP condition 4 score. In step 4, visual processing measures showed a positive association with recall score (*p* = 0.033). See Table 7 for the individual measures.

**Table 7:** Results from the hierarchical regression for CDP condition 4, sway references base of support.

CDP4	Model 1				Model 2				Model 3				Model 4				Model 5			
	*B*	Std. error	*t*	*p*	*B*	Std. error	*t*	*p*	*B*	Std. error	*t*	*p*	*B*	Std. error	*t*	*p*	*B*	Std. error	*t*	*p*
*R* ^2^	0.041				0.064				0.091				0.100				0.109			
Constant	78.610	2.707	29.043	0.000	76.141	3.788	20.103	0.000	63.228	6.408	9.867	0.000	55.647	6.700	8.305	0.000	47.700	8.322	5.732	0.000
DEMOGAGEYEARS	−0.016	0.050	−0.321	0.750	−0.013	0.052	−0.243	0.810	0.015	0.055	0.278	0.784	0.019	0.058	0.337	0.740	0.043	0.058	0.741	0.467
GENDERTYP	−2.563	1.350	−1.898	0.061	−2.938	1.293	−2.272	0.024	−3.092	1.278	−2.420	0.016	−3.000	1.295	−2.317	0.022	−3.074	1.337	−2.299	0.024
TOTAL_TBI	−0.597	0.216	−2.766	0.006	−0.464	0.237	−1.959	0.055	−0.525	0.235	−2.237	0.029	−0.551	0.236	−2.340	0.023	−0.568	0.233	−2.440	0.018
HHIASEMBARRASSEDNEWPEOPLE					−0.209	0.271	−0.772	0.454	−0.186	0.269	−0.692	0.501	−0.180	0.264	−0.681	0.507	−0.150	0.277	−0.539	0.600
HHIASFEELFRUSTRATED					0.181	0.452	0.400	0.692	0.199	0.449	0.443	0.661	0.206	0.436	0.472	0.640	0.177	0.427	0.414	0.681
HHIASDIFFICULTYUNDERSTANDING					0.008	0.540	0.015	0.988	0.032	0.530	0.060	0.953	−0.048	0.535	−0.089	0.930	−0.007	0.514	−0.014	0.989
HHIASFEELHANDICAPPED					0.185	0.408	0.455	0.650	0.312	0.401	0.776	0.438	0.326	0.403	0.810	0.418	0.357	0.401	0.890	0.374
HHIASDIFFICULTYVISITING					−0.053	0.635	−0.084	0.935	−0.098	0.656	−0.149	0.885	−0.153	0.648	−0.237	0.818	−0.163	0.659	−0.247	0.810
HHIASDIFFICULTYINMOVIES					0.069	0.543	0.128	0.901	0.112	0.553	0.203	0.843	0.134	0.538	0.249	0.808	0.174	0.530	0.328	0.748
HHIASARGUMENTSFAMILY					−0.236	0.316	−0.747	0.457	−0.250	0.314	−0.797	0.427	−0.264	0.318	−0.831	0.408	−0.203	0.322	−0.630	0.530
HHIASDIFFICULTYLISTENINGTV					−0.034	0.608	−0.057	0.956	−0.079	0.603	−0.132	0.898	−0.095	0.591	−0.161	0.876	−0.030	0.564	−0.054	0.958
HHIASHAMPERSPERSONALLIFE					−0.529	0.498	−1.062	0.302	−0.402	0.477	−0.843	0.408	−0.378	0.486	−0.777	0.446	−0.354	0.475	−0.746	0.464
HHIASDIFFICULTYRESTAURANT					−0.036	0.610	−0.060	0.954	−0.141	0.584	−0.242	0.813	−0.103	0.584	−0.176	0.864	−0.065	0.576	−0.112	0.913
HHIASPROBLEMWITHHEARING					2.225	1.212	1.836	0.086	2.007	1.209	1.660	0.118	1.852	1.250	1.482	0.161	1.176	1.191	0.988	0.337
BVMTRRECALLTSCORE									0.122	0.057	2.147	0.033	0.122	0.058	2.114	0.036	0.108	0.058	1.880	0.062
BVMTRLEARNINGTSCORE									4.402E-05	0.043	0.001	0.999	0.009	0.044	0.210	0.834	0.002	0.043	0.043	0.965
BVMTRDELAYEDRECALLTSCORE									0.037	0.053	0.691	0.490	0.033	0.053	0.613	0.540	0.036	0.053	0.684	0.494
BVMTRHITRAWSCORE									−0.144	2.207	−0.065	0.949	−0.215	2.162	−0.099	0.922	−0.211	2.079	−0.101	0.920
BVMTRFALSEALARMRAWSCORE									0.502	2.382	0.211	0.835	0.643	2.387	0.269	0.790	0.854	2.333	0.366	0.718
BVMTRDISCRIMINTATIONRAWSCORE									1.116	1.854	0.602	0.553	1.151	1.847	0.623	0.539	1.180	1.800	0.655	0.517
VA_RT_score													−0.733	1.936	−0.379	0.706	−0.807	1.968	−0.410	0.683
VA_RT_inter													4.011	4.119	0.974	0.359	3.703	4.052	0.914	0.387
VA_LT_score													0.814	2.149	0.379	0.708	0.591	2.151	0.275	0.786
VA_LT_inter													3.782	4.767	0.793	0.450	3.919	4.763	0.823	0.434
SCAN3GAPDETECTGRADE																	−0.773	1.860	−0.416	0.681
SCAN3AUDITFIGURECOMBINEDSCORE																	0.254	0.119	2.131	0.051
SCAN3COMPETEWORDCOMBINEDSCORE																	0.091	0.108	0.836	0.410
																	1.176	1.191	0.988	0.337

### 3.8. CDP condition 5 eyes closed with a sway-referenced surface

Table 8 represents the complete hierarchical regression results for the CDP condition 5 score. In step 1 both age (*p* = 0.020) and number of TBI (*p* < 0.001) were shown to be negatively related to CDP condition 5 score. Step 2, showed the absence of difficulty hearing (*p* < 0.001) was associated with a better CDP condition 5 score. In step 4 visual processing measures showed a negative association between recall score (*p* = 0.004). See Table 8 for the individual measures.

**Table 8:** Results from the hierarchical regression for CD5, sway references base of support and occluded vision.

CDP 5	Model 1				Model 2				Model 3				Model 4				Model 5			
	*B*	Std. error	*t*	*p*	*B*	Std. error	*t*	*p*	*B*	Std. error	*t*	*p*	*B*	Std. error	*t*	*p*	*B*	Std. error	*t*	*p*
*R* ^2^	0.059				0.091					0.109			0.113				0.122			
Constant	68.654	2.989	22.971	0.000	63.823	3.839	16.624	0.000	48.588	5.733	8.475	0.000	44.918	6.887	6.522	0.000	35.020	7.781	4.501	0.000
DEMOGAGEYEARS	−0.126	0.053	−2.399	0.020	−0.108	0.055	−1.973	0.057	−0.071	0.055	−1.290	0.205	−0.060	0.058	−1.032	0.311	−0.026	0.059	−0.445	0.659
GENDERTYP	−1.727	1.316	−1.313	0.189	−2.412	1.330	−1.813	0.070	−2.639	1.317	−2.005	0.045	−2.639	1.311	−2.013	0.044	−2.603	1.318	−1.974	0.049
TOTAL_TBI	−0.938	0.249	−3.766	0.000	−0.702	0.267	−2.628	0.013	−0.753	0.266	−2.828	0.008	−0.770	0.267	−2.885	0.007	−0.785	0.263	−2.979	0.005
HHIASEMBARRASSEDNEWPEOPLE					−0.259	0.215	−1.207	0.230	−0.243	0.222	−1.093	0.279	−0.238	0.219	−1.087	0.280	−0.206	0.220	−0.938	0.351
HHIASFEELFRUSTRATED					0.275	0.534	0.514	0.614	0.303	0.537	0.564	0.581	0.291	0.528	0.552	0.588	0.251	0.507	0.495	0.626
HHIASDIFFICULTYUNDERSTANDING					0.198	0.509	0.389	0.700	0.194	0.505	0.384	0.703	0.163	0.526	0.309	0.760	0.235	0.511	0.459	0.649
HHIASFEELHANDICAPPED					−0.611	0.480	−1.272	0.209	−0.478	0.464	−1.031	0.305	−0.438	0.469	−0.934	0.353	−0.394	0.464	−0.849	0.398
HHIASDIFFICULTYVISITING					0.347	0.534	0.651	0.521	0.328	0.535	0.613	0.545	0.301	0.533	0.565	0.577	0.280	0.538	0.521	0.607
HHIASDIFFICULTYINMOVIES					−0.030	0.483	−0.062	0.951	−0.054	0.482	−0.111	0.912	−0.034	0.482	−0.070	0.945	−0.020	0.473	−0.043	0.966
HHIASARGUMENTSFAMILY					−0.377	0.406	−0.929	0.363	−0.353	0.380	−0.929	0.359	−0.364	0.381	−0.955	0.346	−0.294	0.381	−0.772	0.445
HHIASDIFFICULTYLISTENINGTV					−0.295	0.501	−0.590	0.563	−0.353	0.500	−0.706	0.489	−0.362	0.496	−0.730	0.474	−0.281	0.465	−0.605	0.550
HHIASHAMPERSPERSONALLIFE					−0.519	0.622	−0.835	0.422	−0.406	0.629	−0.646	0.532	−0.396	0.636	−0.623	0.547	−0.358	0.627	−0.572	0.580
HHIASDIFFICULTYRESTAURANT					−0.205	0.473	−0.433	0.667	−0.289	0.505	−0.572	0.573	−0.304	0.521	−0.584	0.566	−0.284	0.519	−0.547	0.591
HHIASPROBLEMWITHHEARING					4.138	1.036	3.995	0.000	3.948	1.051	3.757	0.000	3.867	1.061	3.645	0.001	3.283	1.065	3.081	0.003
BVMTRRECALLTSCORE									0.162	0.058	2.825	0.005	0.166	0.058	2.853	0.004	0.151	0.058	2.593	0.010
BVMTRLEARNINGTSCORE									−0.003	0.042	−0.070	0.944	0.005	0.043	0.112	0.911	0.001	0.043	0.013	0.989
BVMTRDELAYEDRECALLTSCORE									−0.062	0.060	−1.035	0.302	−0.068	0.060	−1.135	0.258	−0.069	0.060	−1.143	0.255
BVMTRHITRAWSCORE									2.305	2.079	1.109	0.274	2.194	2.137	1.027	0.312	2.091	2.193	0.954	0.349
BVMTRFALSEALARMRAWSCORE									−1.836	2.520	−0.729	0.474	−1.800	2.539	−0.709	0.486	−1.608	2.566	−0.626	0.538
BVMTRDISCRIMINTATIONRAWSCORE									−0.494	1.984	−0.249	0.806	−0.438	2.014	−0.218	0.830	−0.373	2.057	−0.181	0.858
VA_RT_score													−0.947	2.224	−0.426	0.673	−0.847	2.201	−0.385	0.702
VA_RT_inter													4.362	4.303	1.014	0.340	4.028	4.272	0.943	0.372
VA_LT_score													1.761	2.258	0.780	0.441	1.454	2.263	0.642	0.525
VA_LT_inter													−1.552	3.753	−0.414	0.683	−1.223	3.668	−0.333	0.742
SCAN3GAPDETECTGRADE																	−0.441	1.696	−0.260	0.795
SCAN3AUDITFIGURECOMBINEDSCORE																	0.158	0.098	1.606	0.112
SCAN3COMPETEWORDCOMBINEDSCORE																	0.233	0.119	1.962	0.060

### 3.9. CDP condition 6 eyes open with sway-referenced surface and visual surrounds

Table 9 represents the complete hierarchical regression results for the CDP 6 score. In step 1 both age (*p* = 0.021) and number of TBI (*p* = 0.016) were shown to be negatively related to CDP condition 6 score. In step 2 the absence of difficulty hearing (*p* < 0.001) was associated with better CDP condition 6 score. Step 3 revealed recall score (*p* = 0.004) showed a positive relation. In step 4 visual processing measure showed a negative association with learning score (*p* = 0.011). See Table 9 for the individual measures.

**Table 9:** Results from the hierarchical regression for CDP condition 6, sway references base of support and vision.

CDP 6	Model 1				Model 2				Model 3				Model 4				Model 5			
	*B*	Std. error	*t*	*p*	*B*	Std. error	*t*	*p*	*B*	Std. error	*t*	*p*	*B*	Std. error	*t*	*p*	*B*	Std. error	*t*	*p*
*R* ^2^	0.046				0.080				0.113				0.120				0.128			
Constant	68.716	3.077	22.331	0.000	64.929	4.274	15.190	0.000	51.166	7.334	6.977	0.000	46.428	7.624	6.090	0.000	35.666	8.934	3.992	0.000
DEMOGAGEYEARS	−0.126	0.054	−2.332	0.021	−0.118	0.055	−2.127	0.035	−0.072	0.056	−1.289	0.200	−0.057	0.060	−0.952	0.345	−0.015	0.059	−0.260	0.795
GENDERTYP	−2.803	1.512	−1.853	0.064	−3.379	1.501	−2.252	0.025	−3.714	1.475	−2.517	0.012	−3.753	1.461	−2.569	0.010	−3.655	1.458	−2.506	0.012
TOTAL_TBI	−0.678	0.275	−2.461	0.016	−0.456	0.275	−1.656	0.101	−0.532	0.278	−1.914	0.060	−0.552	0.278	−1.984	0.051	−0.572	0.276	−2.073	0.042
HHIASEMBARRASSEDNEWPEOPLE					−0.008	0.450	−0.019	0.986	−0.009	0.455	−0.021	0.984	−0.007	0.456	−0.015	0.989	0.030	0.462	0.065	0.950
HHIASFEELFRUSTRATED					−0.014	0.701	−0.020	0.984	0.066	0.679	0.097	0.924	0.058	0.669	0.086	0.933	0.016	0.669	0.025	0.981
HHIASDIFFICULTYUNDERSTANDING					−0.337	0.611	−0.552	0.587	−0.288	0.585	−0.492	0.626	−0.338	0.599	−0.564	0.578	−0.264	0.588	−0.450	0.657
HHIASFEELHANDICAPPED					−0.519	0.625	−0.830	0.417	−0.301	0.581	−0.518	0.608	−0.224	0.582	−0.384	0.704	−0.165	0.587	−0.281	0.781
HHIASDIFFICULTYVISITING					0.222	0.943	0.236	0.820	0.133	0.948	0.140	0.892	0.094	0.956	0.098	0.924	0.065	0.972	0.067	0.949
HHIASDIFFICULTYINMOVIES					0.337	0.540	0.624	0.537	0.370	0.554	0.667	0.511	0.400	0.548	0.729	0.472	0.422	0.538	0.783	0.440
HHIASARGUMENTSFAMILY					−0.238	0.688	−0.346	0.739	−0.213	0.646	−0.330	0.750	−0.234	0.642	−0.364	0.725	−0.146	0.656	−0.223	0.829
HHIASDIFFICULTYLISTENINGTV					−0.379	0.641	−0.591	0.566	−0.423	0.625	−0.677	0.511	−0.443	0.619	−0.717	0.487	−0.352	0.598	−0.588	0.566
HHIASHAMPERSPERSONALLIFE					−0.699	0.614	−1.140	0.270	−0.538	0.575	−0.935	0.360	−0.529	0.569	−0.929	0.363	−0.478	0.561	−0.852	0.403
HHIASDIFFICULTYRESTAURANT					0.326	0.514	0.635	0.529	0.146	0.549	0.266	0.792	0.112	0.569	0.196	0.846	0.142	0.570	0.249	0.806
HHIASPROBLEMWITHHEARING					3.797	1.030	3.685	0.000	3.809	1.030	3.698	0.000	3.689	1.042	3.540	0.000	2.957	1.056	2.801	0.005
BVMTRRECALLTSCORE									0.224	0.074	3.009	0.004	0.228	0.075	3.034	0.004	0.209	0.076	2.738	0.009
BVMTRLEARNINGTSCORE									−0.128	0.050	−2.574	0.011	−0.117	0.050	−2.368	0.018	−0.123	0.049	−2.498	0.013
BVMTRDELAYEDRECALLTSCORE									−0.022	0.070	−0.313	0.755	−0.029	0.070	−0.416	0.679	−0.031	0.069	−0.452	0.653
BVMTRHITRAWSCORE									0.038	2.396	0.016	0.988	−0.120	2.415	−0.050	0.961	−0.148	2.464	−0.060	0.953
BVMTRFALSEALARMRAWSCORE									1.135	2.703	0.420	0.678	1.141	2.681	0.425	0.674	1.354	2.676	0.506	0.617
BVMTRDISCRIMINTATIONRAWSCORE									1.776	2.187	0.812	0.424	1.830	2.202	0.831	0.414	1.849	2.225	0.831	0.414
VA_RT_score													−0.887	2.959	−0.300	0.768	−0.813	2.970	−0.274	0.788
VA_RT_inter													7.226	2.963	2.438	0.017	6.862	2.877	2.385	0.018
VA_LT_score													1.905	2.593	0.735	0.469	1.606	2.653	0.605	0.551
VA_LT_inter													−3.335	4.860	−0.686	0.506	−2.975	4.811	−0.618	0.548
SCAN3GAPDETECTGRADE																	−1.495	2.141	−0.698	0.489
SCAN3AUDITFIGURECOMBINEDSCORE																	0.197	0.121	1.635	0.111
SCAN3COMPETEWORDCOMBINEDSCORE																	0.262	0.136	1.919	0.067

## 4. Discussion

The goal of this study was to determine the relationships between sensory function and postural balance among current and former combat-exposed service members, with and without a history of mTBI(s). Balance is dependent on the ability to combine and process sensory information, identifying the fidelity of these signals and using this information to adjust the weighting of the sensory information (12). This study reinforces that postural balance is a complex control problem that utilizes multiple sensory systems and requires the ability to successfully process multiple inputs at the executive processing level.

In general, individuals with TBI can reliably maintain postural stability (as evidenced by high CDP scores for Condition 1 in Table 1) and ambulate at similar speeds successfully when sensory input from vision, proprioception, or vestibular systems are unperturbed. However, individuals with TBI have more difficulty when adjustments in the weighting of these sensory inputs are required due to various experimental perturbations; swaying surrounding or base of support, or the occlusion of vision.

The most consistent feature across regression analyses was that sensory disruptions (vision, vestibular, or somatosensory) and subsequent lower balance assessment outcomes (via CDP 2–6 scores) were associated with the number of TBIs reported (29). Additionally, females appear to have more difficulty keeping their balance when proprioception is unreliable (e.g., on a swaying surface) than males. Counterintuitively, age shows to be associated with faster walking speed, however as expected, older participants had lower scores on balance assessments (CDP 2, 5 & 6 and the Composite score).

Surprisingly, variance accounted for by the combination of all demographic and sensory acuity/processing items only attributed 10.9 and 14.0% of the total variability in the balance and gait outcomes. While this may seem limited, many factors affect gait and balance not accounted for in these models. In general, more associations were found between the visual and auditory processing measures compared to specific hearing and vision impairments, more in-depth discussion follows below.

Deficits in hearing as assessed by the self-reported hearing difficulties on the hearing handicap questionnaire (HHIA-S) showed associations with measures of balance (CDP, except CDP1) and gait. Participants who indicated to have problems with hearing showed to have slower walking speeds and lower balance scores (CDP composite and CDP2-6). Additionally, ‘difficulty understanding movies’ showed an association with slower walking speeds. These findings are consistent with previous studies by Viljanen et al. (21) showing that women with poorer hearing have poorer postural control and higher fall risk. Authors have postulated this to be related to the anatomical location of the vestibular system to the auditory system, along with their shared vestibulocochlear nerve, vascular supply, similar mechanosensory receptor hair cells, which detect sound, head movements, and orientation in space, and therefore with balance (21).

Auditory processing (SCAN3) was shown to be associated with gait speed and balance (composite score and CDP 1–3). The ability to better distinguish words from noise (SCAN audio figure score) was associated with faster gait speeds and better CDP composite score, so in general better gait and balance. Additionally, the ability to better repeat word pairs (compete word score) presented to be associated with faster gait speeds and better balance scores while proprioception was not perturbed (i.e., standing surface was stable in CDP1-3). So, when proprioception does not have to be re-weighted, but vision may or may not be perturbed, participants with a better ability to recall word pairs are shown to be better at maintaining balance with all sensory intact (CDP 1), or occluded or perturbed vision (CDP 2 and 3). In previous literature auditory processes have been shown to slow down gait; elderly stop walking when talking (30), and affect foot placement; stroke survivors lag auditory cues for foot falls (31). These findings suggest that the ability to inhibit noise, remember word pairs, and process auditory stimuli benefits gait speed and balance.

Visual acuity (VA) only showed an association with balance when vision and proprioception were sway-referenced, participants with impaired vision on the right had worse balance scores on the CDP6. No associations were found for visual acuity and walking speed, nor the other balance measures. In previous literature relationships of visual acuity among other visual measures and self-reported ability to walk a quarter of a mile or walking up 10 steps (20) were shown in the aging population (70–79). The lack of associations with vision, gait, and balance in this study could be caused by multiple factors. One of these factors may be that other visual functions are more important for balance and gait, like peripheral vision or spatial relations. Secondly, the demographics (age range 22–71) of this cohort did only show impaired vision (20/40 met) of 4.2% in the right eye and 2.7% in the left, where Swenor et al. found 7.4% to have vision impairment when looking with both eyes. Additionally, literature has reported various outcomes on the associations between vision, balance, and gait measures. Many studies have indicated the ability to detect movements (32, 33) or having visual blur (34, 35) affects balance. Visual acuity may not be directly related to gait speed or balance, it has been identified as a risk factor for falls (36–40), however, when adjusting for age these associations were not found (41–48).

Visual processing (BVMT) showed a more complex association with gait speed and balance. The ability to immediately recall a figure was associated with faster gait speed, while a delayed recall was associated with slower gait speed. Doi et al. showed that better visual memory was associated with faster gait speed, especially in participants with mild cognitive impairment (49). This is in agreement with literature showing people slow down (15, 50) and attentional costs increase (51–53) when walking to visual targets, and sway area (an often used balance measure) increases when eyes are closed (54). However, better delayed recall (after a 25-min delay) is associated with slower gait speeds. This association of slower gait speed with delayed memory as increased cortical attention/demand is required to recall, therefore visual processing requires greater attentional resources (55). Better general balance scores (CDP composite) and balance when vision was compromised or occluded and proprioception was compromised (CDP 5 and 6) are associated with better direct visual recall and better balance when vision was occluded (CDP 2) showed associations with delayed recall. Indicating that participants who rely on visuo-spatial memory when visual information is crude may prevent them from indicating what sensory information is reliable and upregulate those systems, affecting their ability to maintain balance. This confirms that visual processing is more important when proprioception is compromised.

### 4.1. Limitations

A large proportion of the non-TBI participants (53.85%) had relatively low SOT-composite scores (less than 75). In a manufacturer’s stated normative data set only 20% of ‘normal’ individuals had composite scores below 75. The higher proportion in our sample may be due to comorbidities, including chronic pain, PTSD, and sleep apnea in Veterans and Service Members (56), which previous preliminary analyses have linked to lower SOT-composite scores in Veterans and Service Members (9). Given that our sample had all served in the military and was predominantly male, results may not generalize to civilian or female populations and therefore, a similar analysis may be performed with a general public control group in the future. Therefore, relationships between sensory and processing deficits and gait and balance may be underestimated. In the future, similar analysis may be done on a population with greater balance and gait deficits and or when this cohort ages more.

Additionally, a large proportion of the data had to be imputed due to missing values. However, imputing missing values is known to reduce bias and improve efficiency over complete case analysis over excluding missing data (57, 58).

## 5. Conclusion

In general, individuals with TBI maintained postural stability and ambulation as well as their healthy counterparts, likely showing an ability to adapt to their sensory impairments (shown in acuity and processing outcomes). However, balance deficits may be unmasked when re-weighting inputs is required due to sensory disruption (e.g., during light adaptation to a dimly lit room), and may have greater consequences with more frequent exposure to TBIs. Our findings reinforce that sensory processing (rather than acuity) is more associated with negative balance and gait outcomes and potential increases in fall risk.

## Data availability statement

The data analyzed in this study is subject to the following licenses/restrictions: the CENC/LIMBIC data board has to approve data availability. Requests to access these datasets should be directed to Sudeep Karki Sudeep Karki@vcuhealth.org.

## Ethics statement

The studies involving humans were approved by Richmond VAMC Research and Development Committee. The studies were conducted in accordance with the local legislation and institutional requirements. The participants provided their written informed consent to participate in this study.

## Author contributions

SV: conceptualize, analyze, and write a first draft. RP: conceptualize analysis, statistical advice, and proofread. LF: EEG data analysis and proofread. AA, KS, SS, EW, AS, JT, and WW: conceptualize analysis, methods, and proofreading. All authors contributed to the article and approved the submitted version.

## Funding

This work was supported by the Assistant Secretary of Defense for Health Affairs and endorsed by the Department of Defense, through the Psychological Health/Traumatic Brain Injury Research Program Long-Term Impact of Military-Relevant Brain Injury Consortium (LIMBIC) Award/W81XWH-18-PH/TBIRP-LIMBIC under Awards Nos. W81XWH1920067 and W81XWH-13-2-0095, and by the U.S. Department of Veterans Affairs Awards No. I01 CX002097, I01 CX002096, I01 HX003155, I01 RX003444, I01 RX003443, I01 RX003442, I01 CX001135, I01 CX001246, I01 RX001774, I01 RX 001135, I01 RX 002076, I01 RX 001880, I01 RX 002172, I01 RX 002173, I01 RX 002171, I01 RX 002174, and I01 RX 002170. The U.S. Army Medical Research Acquisition Activity, 839 Chandler Street, Fort Detrick MD 21702-5014 is the awarding and administering acquisition office. Opinions, interpretations, conclusions, and recommendations are those of the author and are not necessarily endorsed by the Department of Defense.

## Conflict of interest

The authors declare that the research was conducted in the absence of any commercial or financial relationships that could be construed as a potential conflict of interest.

## Publisher’s note

All claims expressed in this article are solely those of the authors and do not necessarily represent those of their affiliated organizations, or those of the publisher, the editors and the reviewers. Any product that may be evaluated in this article, or claim that may be made by its manufacturer, is not guaranteed or endorsed by the publisher.
